# Compression and stretch sensitive submucosal neurons of the porcine and human colon

**DOI:** 10.1038/s41598-020-70216-6

**Published:** 2020-08-14

**Authors:** Anna Katharina Filzmayer, Kristin Elfers, Klaus Michel, Sabine Buhner, Florian Zeller, Ihsan Ekin Demir, Jörg Theisen, Michael Schemann, Gemma Mazzuoli-Weber

**Affiliations:** 1 Institute for Physiology and Cell Biology, University of Veterinary Medicine, Foundation, Hannover, Germany; 2 Chair of Human Biology, Technical University of Munich, Freising, Germany; 3 Academic Hospital Freising, Freising, Germany; 4 University Hospital Rechts der Isar, Technical University of Munich, Munich, Germany; 5 Visceral and Thoracic Surgery, Academic Hospital Erding, Erding, Germany

**Keywords:** Physiology, Gastroenterology

## Abstract

The pig is commonly believed to be a relevant model for human gut functions—however, there are only a few comparative studies and none on neural control mechanisms. To address this lack we identified as one central aspect mechanosensitive enteric neurons (MEN) in porcine and human colon. We used neuroimaging techniques to record responses to tensile or compressive forces in submucous neurons. Compression and stretch caused Ca-transients and immediate spike discharge in 5–11% of porcine and 15–24% of human enteric neurons. The majority of these MEN exclusively responded to either stimulus quality but about 9% responded to both. Most of the MEN expressed choline acetyltransferase and substance P; nitric oxide synthase-positive MEN primarily occurred in distal colon. The findings reveal common features of MEN in human and pig colon which we interpret as a result of species-independent evolutionary conservation rather than a specific functional proximity between the two species.

## Introduction

The enteric nervous system (ENS), which is integrated into the wall of the gastrointestinal tract (GIT) from the oesophagus to the anal sphincter, enables the GIT to generate reflex activity independent from central influences^1–3^. The ENS is organized in two complex neuronal networks called the submucosal plexus (SMP) and the myenteric plexus (MP). In both, the pig and human the SMP consists of two layers interconnected by interganglionic nerve fiber tracts^4,5^. The SMP regulates mostly epithelial functions, such as secretion and absorption, as well as blood flow, cell proliferation and immune responses.

The distinct parts of the SMP are called inner SMP (ISMP) and outer SMP (OSMP) with the ISMP being located closely to the lamina muscularis mucosae, whereas the OSMP is located on the luminal side of the circular muscle layer^6^. In the porcine and human colon mainly neurons in the ISMP predominantly project to the mucosa^7–9^, and hence likely regulate epithelial functions^10^.

Enteric neurons can be activated by various stimuli, including chemical stimuli as well as mechanical distortion^11–17^. During muscle contraction and relaxation enteric neurons are constantly exposed to distorting forces^16,18^. Remarkably, even enteric neurons classically defined as interneurons or motoneurons are mechanosensitive, suggesting that mechanosensitive enteric neurons (MEN) are multifunctional^14,15,19^. With intracellular recording techniques it has been shown that MEN in the MP respond to distension as well as to mucosal distortion^20–22^. An important step forward was the use of imaging techniques, as they allowed to record simultaneously from a larger set of neurons and were also a prerequisite to study enteric neurons in larger animals^14–17,23,24^. Using von Frey hair probing and intraganglionic volume injection, compression sensitive MEN have been identified in the MP of the guinea pig gastric corpus, ileum and colon^14,16,23^ as well as in mouse ileum and colon^15^. Furthermore, von Frey hair probing was used to identify compression sensitive MEN in isolated cultured myenteric neurons from human intestinal samples^23^. In addition, in myenteric neurons of the guinea pig gastric corpus, ganglionic stretch was used to study neuronal sensitivity to tensile forces^16^. Depending on the region studied, 15–27% of all myenteric neurons were identified as MEN^14–16^.

Our hypothesis is that MEN also exist in the ISMP of the porcine and human colon. This is supported by studies that showed nerve-mediated chloride secretion in laboratory animal models in response to distension of mucosal-submucosal preparations^25–28^. Data on the mechanosensitivity of submucosal neurons are scarce. Neurons in the SMP, referred to as intrinsic primary afferent neurons, respond to mechanical stimulation of the mucosa^29,30^. However, these are second-order neurons, as the sensory cell transducing the stimulus is the enterochromaffin cell, which activates serotonin (1P) receptors on submucosal primary afferent neurons by releasing serotonin^29^.

Unlike for the MP, it remains unknown whether neurons in the SMP respond to mechanical stimulation. Therefore, our study aimed to identify mechanosensitive neurons in the SMP of the pig and human colon. We used this plexus in the colon as disturbances of sensory-motor pathways here are likely to be responsible for functional disorders associated with diarrhoea or constipation. The choice of the two species is related to the clinical relevance as well as to the widely accepted notion that the pig is a good model for human based on anatomical, nutritional and physiological similarities^31–36^. We used voltage and Ca^2+^ imaging to record response from submucous neurons, located in the ISMP to compressive and tensile forces. In addition, we investigated the basic neurochemical code of porcine MEN with respect to their choline acetyltransferase (ChAT)-, substance P (SP)- and nitric oxid synthase (NOS)-immunoreactivity. Our study revealed for the first time in the ISMP neurons, which responded specifically to compressive or tensile forces. The percentage of MEN as well as their spike frequency was strikingly comparable between pig and human.

## Results

### In the porcine ISMP there are MEN, which reproducibly respond to compression and stretch

As expected, we did not detect an age-dependent responsiveness of MEN as the juvenile pigs were between 7 and 24 weeks old. We therefore pooled data from animals of different age.

**Figure 1:**
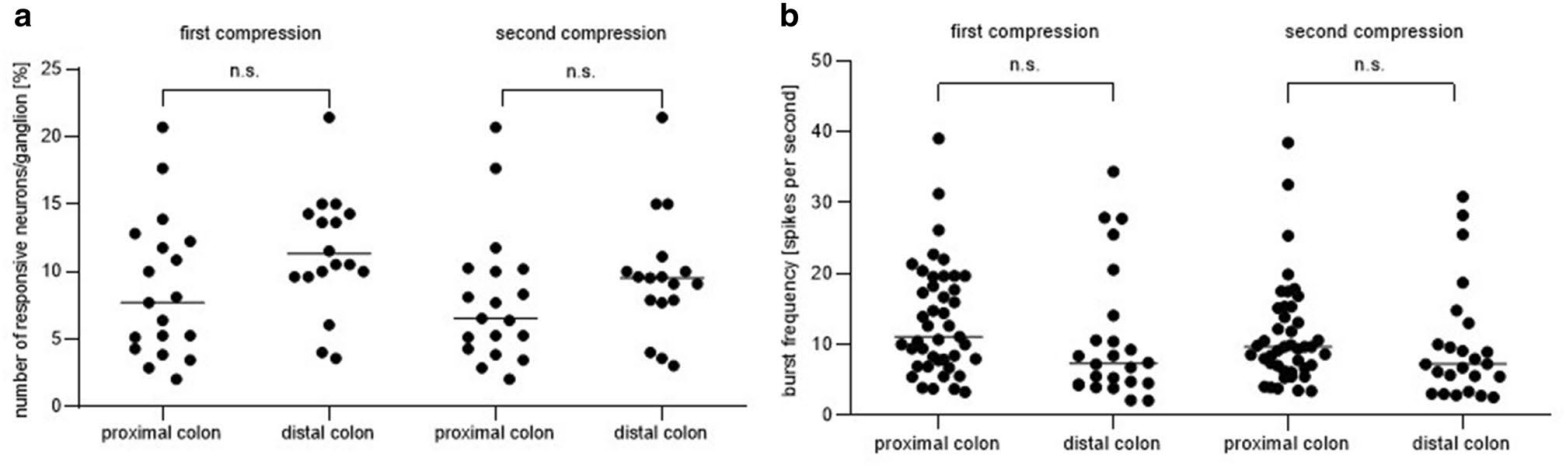
Number of responsive neurons/ganglion (**a**) and burst frequency (**b**) after neuronal compression by intraganglionic volume injection in submucosal neurons of the porcine proximal and distal colon for a first and second compression stimulus, respectively. Number of responsive cells/ganglion and burst frequency were not significantly different between the two localisations investigated for a first and a second compression stimulus (unpaired *t* test and Mann–Whitney). Proximal colon n = 15/19/730; distal colon n = 11/12/302.

Neuronal responses to mechanical compression by intraganglionic injection could be reproducibly recorded from neurons of the proximal and distal colon which hence were defined as compression sensitive. Supplementary Video 1 shows an example of intraganglionic injection into a porcine submucosal ganglion of the proximal colon containing compression sensitive MEN responding to this stimulus with action potential discharge. The number of neurons per ganglion responding as well as their burst frequencies were not statistically different between the two localisations investigated for a first and second compression stimulus (Fig. 1).

**Figure 2:**
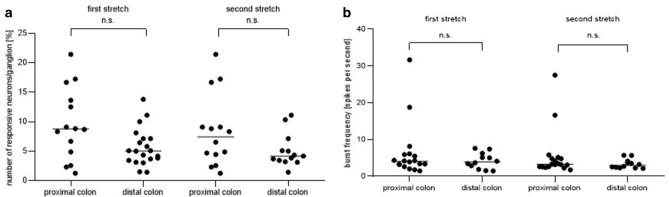
Number of responsive neurons/ganglion (**a**) and burst frequency (**b**) after bidirectional stretch in submucosal neurons of the porcine proximal and distal colon for a first and second stretch stimulus, respectively. Number of responsive cells/ganglion and burst frequency were not significantly different between the two localisations investigated for a first and a second stretch stimulus (Mann–Whitney test). Proximal colon n = 10/13/437; distal colon n = 10/13/488.

Stretch led to reproducible responses in submucous neurons from the proximal and distal colon, respectively. Neither the number of neurons per ganglion nor burst frequencies were statistically different between the two colonic localisations investigated for a first and a second stretch stimulus (Fig. 2). On average 7.9% and 10.4% of the total ISMP porcine neurons were compression sensitive in the proximal and distal colon, respectively. 7.7% and 6.3% of the total ISMP porcine neurons were stretch sensitive in the proximal and distal colon, respectively.

### Correlation of stretch led changes in cell surface with neuronal responses in porcine colon

**Figure 3:**
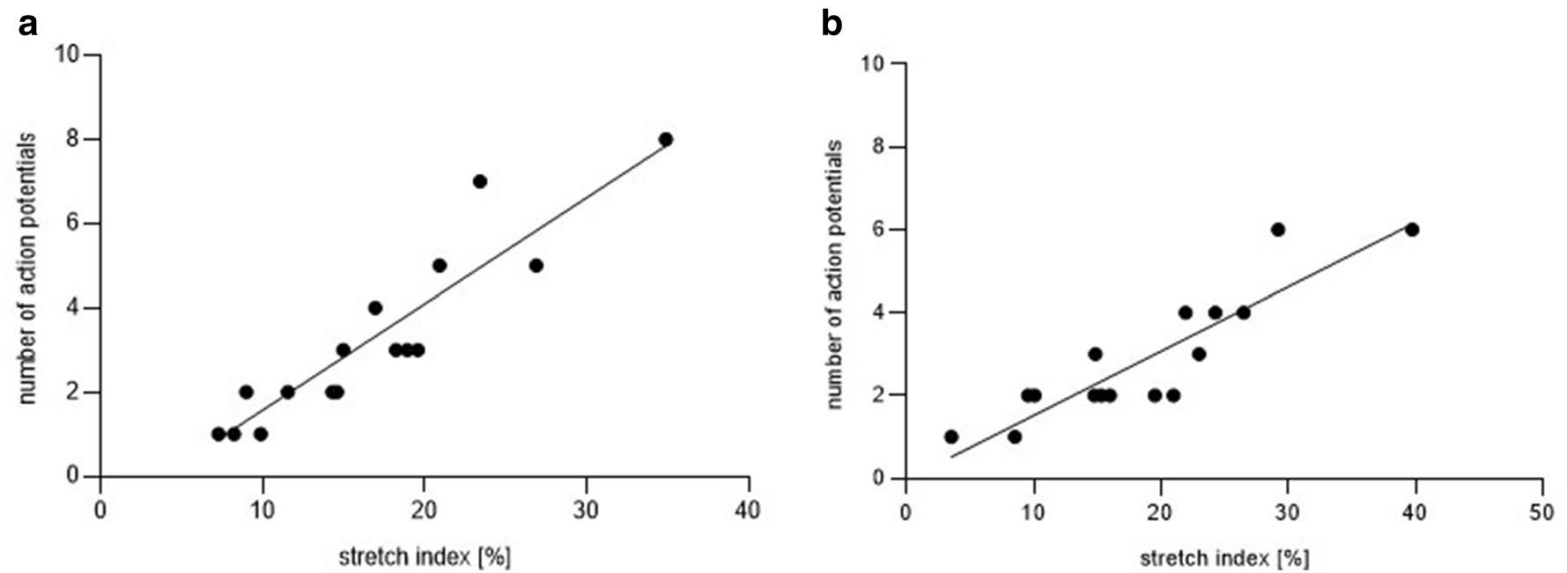
Linear regression of strength of stretch stimulus (given as stretch index) with corresponding number of action potentials (AP) in submucosal neurons of the porcine proximal colon (**a**) (stretch index = (15.88 ± 7.51), number of AP = (3.06 ± 0.48); r^2^ = 0.86, *P* < 0.001) and distal colon (**b**) (stretch index = (18.00 ± 9.07), number of AP = (2.77 ± 1.56); r^2^ = 0.81, *P* < 0.001). n = 4/4/18 (proximal colon), n = 6/6/17 (distal colon); means ± SD. Calculations are only given when significance was obtained by linear regression. Regression line is only presented when Spearman’s r > 0.50.

Stretch led to changes in porcine neuronal area. This was analysed for 18 randomly chosen MEN of the proximal colon originating from four ganglia from four pigs (4/4/18) and 17 MEN of the distal colon originating from six ganglia from six pigs (6/6/17). It has been demonstrated that these MEN are stretch sensitive in ultrafast neuroimaging experiments before. Our results indicated that proximal colon MEN were elongated by 16.3 ± 9.1% and distal colon MEN by 18.00 ± 9.07% on average by the applied stretch. Correlation analysis showed that a higher stretch index induced significantly higher number of action potentials in MEN of both intestinal localisations (Fig. 3).

### Neuronal responsiveness to compression and stretch: paired experiments

**Figure 4:**
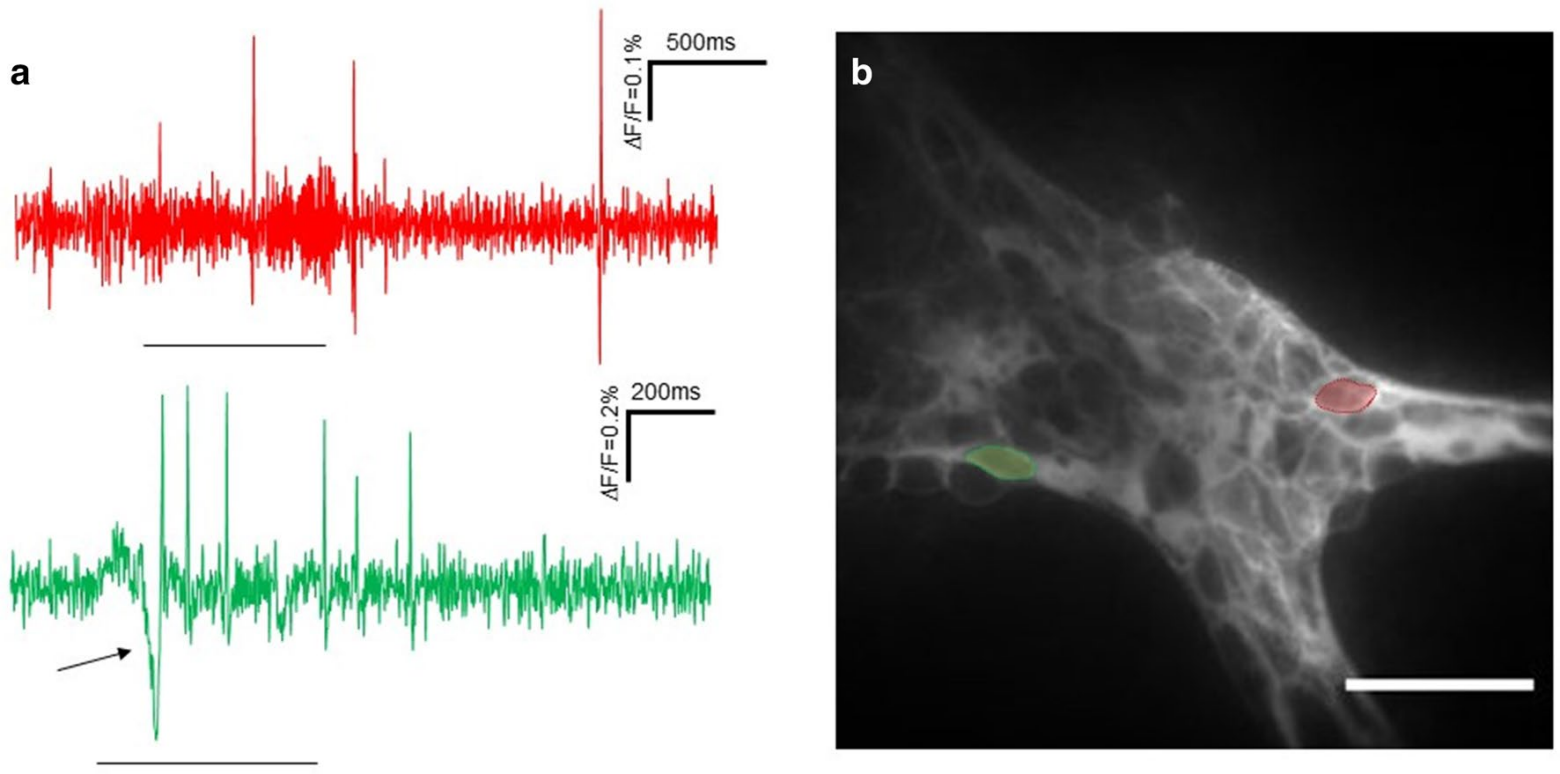
Double stimulus. (**a**) Shows the responses of two mechanosensitive enteric neurons (MEN) from the same submucosal ganglion of the porcine proximal colon to stretch by bidirectional ganglionic stretch (red trace) and to compression by intraganglionic volume injection (green trace). The two traces in (**a**) correspond to the two neurons marked with red and green dotted lines in (**b**). The bar below the traces indicates the onset and end of the stretch and volume injection, respectively. Within the green trace, the first deflection after the compression stimulus is the mechanical artefact due to the stimulus (marked by arrow). Scale bar in (**b)** = 100 µm.

In five ganglia of proximal colon tissues from five pigs we recorded neuronal responses to both stimuli in the same ganglion: ganglionic stretch and intraganglionic volume injection (Fig. 4). 5.6 [3.0/9.3]% of neurons per ganglion responded with a burst frequency of 3.7 [1.4/4.5] spikes per second to the stretch stimulus, whereas 9.1 [5.6/18.8]% of neurons fired action potentials with a burst frequency of 8.6 [4.8/9.7] spikes per second after compression. Of 34 MEN identified in the examined ganglia 10 responded only to the stretch stimulus and 21 only to compression stimulus. The remaining three neurons responded to both stimuli with a burst frequency of 7.2 [3.5/7.4] and 8.7 [5.1/9.3] spikes per second to the stretch and compression stimulus, respectively.

### Porcine MEN are predominantly ChAT/SP immunoreactive

Firstly, because no consistent data exist on the general proportion of NOS immunoreactive neurons among the total number of neurons in the porcine ISMP, we determined this proportion in 75 ganglia of the proximal and distal colon originating from three pigs from age group 1 for each colonic localisation. On average, in porcine ganglia 76.8 ± 64.7 and 76.1 ± 57.6 neurons per ganglion were counted in the proximal and distal colon, respectively. From these neurons 10.3 [5.1/17.1]% and 29.5 [20.0/40.0]% showed immunoreactivity for NOS in the proximal and distal colon.

After determining the proportion of NOS immunoreactive neurons in the porcine colon ISMP we studied the chemical code of compression sensitive and stretch sensitive MEN.

**Figure 5:**
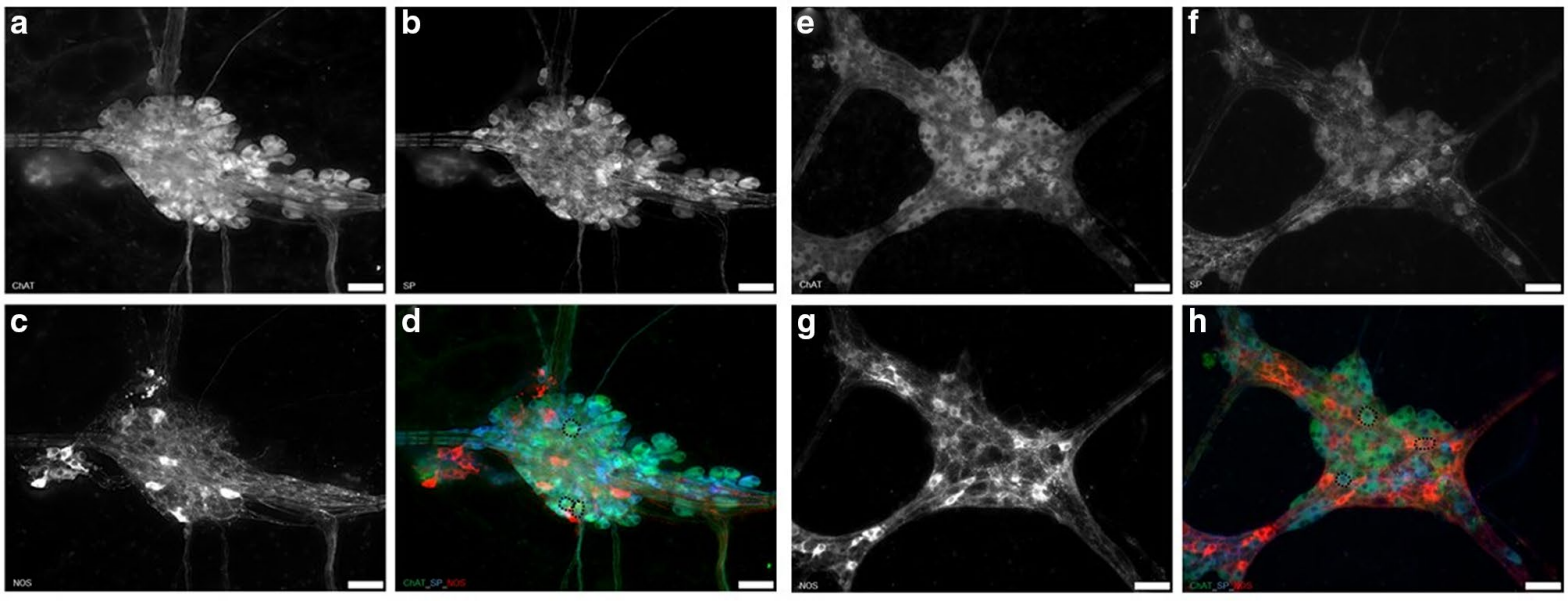
Immunohistochemical staining of mechanosensitive submucosal neurons of the porcine proximal and distal colon. Multilabelled ganglion of the ISMP of the porcine proximal colon (**a**–**d**) and distal colon (**e**–**h**). Immunoreactive neurons were found for ChAT (**a**, **e**), SP (**b**, **f**) and NOS (**c**, **g**). Overlay shows neurons expressing ChAT only, co-expressing ChAT and SP and exclusively expressing NOS (**d**, **h**); three neurons identified as mechanosensitive neurons in neuroimaging experiments before, are encircled by dotted lines. (**d**, **h**). Scale bar = 50 µm. *ISMP* inner submucous plexus.

**Figure 6:**
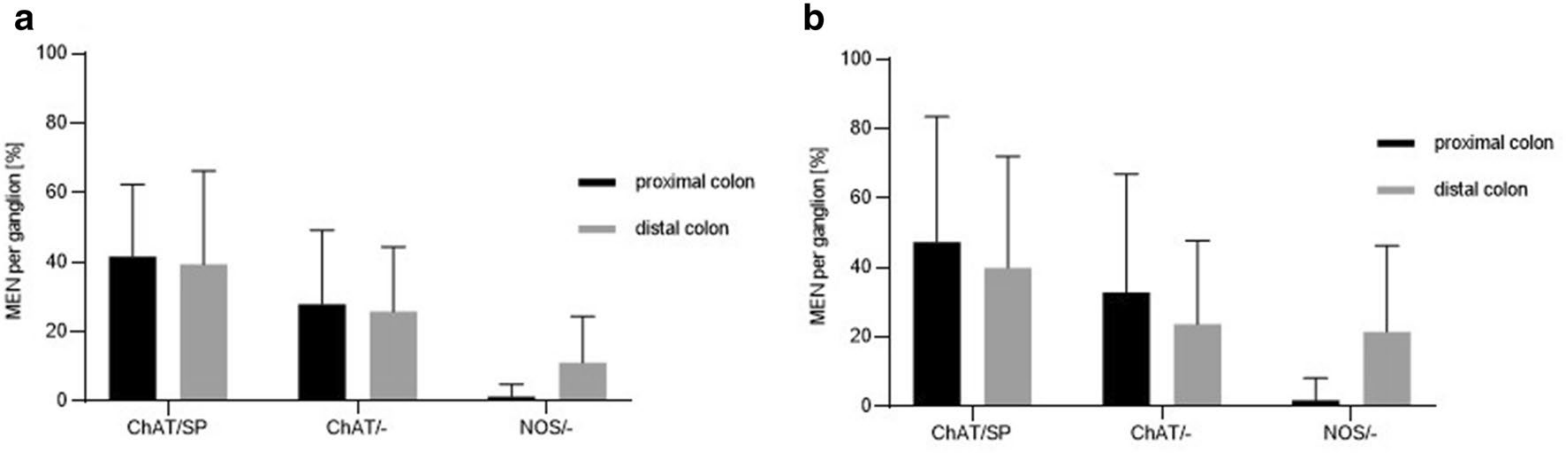
Immunohistochemically identified subpopulations of compression sensitive (**a**) and stretch sensitive (**b**) submucosal neurons of the porcine proximal and distal colon. Majority of neurons identified as compression or stretch sensitive within neuroimaging experiments showed positive staining for ChAT and SP or ChAT only in both, proximal and distal colon. Proportion of NOS positive mechanosensitive neurons was numerically higher in the distal compared to the proximal colon for both mechanical stimuli.

In the proximal colon 69.0% of the identified compression sensitive MEN were cholinergic and 64.0% of these neurons additionally expressed SP (ChAT/SP). No MEN solely immunoreactive for SP were detected, but SP-stained MEN were always coexpressed ChAT immunoreactivity. 2.8% of compression sensitive MEN in proximal colon were exclusively immunoreactive for NOS (NOS/-). In the distal colon 64.0% of the examined compression sensitive MEN were immunoreactive for ChAT antibody with 60.4% additionally expressing SP. As in the proximal colon, no MEN solely expressing SP staining could be detected in the distal colon. NOS antibody immunoreactivity was observed in 12.0% of the compression sensitive MEN in the distal colon (Figs. 5, 6). Similar proportions were found in the two age groups. In the proximal colon 71.4% of stretch sensitive MEN (40 out of 56) were ChAT immunoreactive with 62.5% (25 out of 40 neurons) additionally expressing SP (ChAT/SP). Only one mechanosensitive neuron (1.79%) was NOS immunoreactive. In the distal colon, ChAT immunoreactivity was found in 63.4% of the stretch sensitive MEN (26 out 41) with 61% also showing SP antibody staining. 19.1% of the stretch sensitive MEN in the distal colon proved to be exclusively NOS antibody immunoreactive (Figs. 5, 6). As in the proximal colon we did not find age related differences in the chemical code of compression and stretch sensitive MEN.

### Neurons in the human ISMP respond to mechanical stimulation

**Figure 7:**
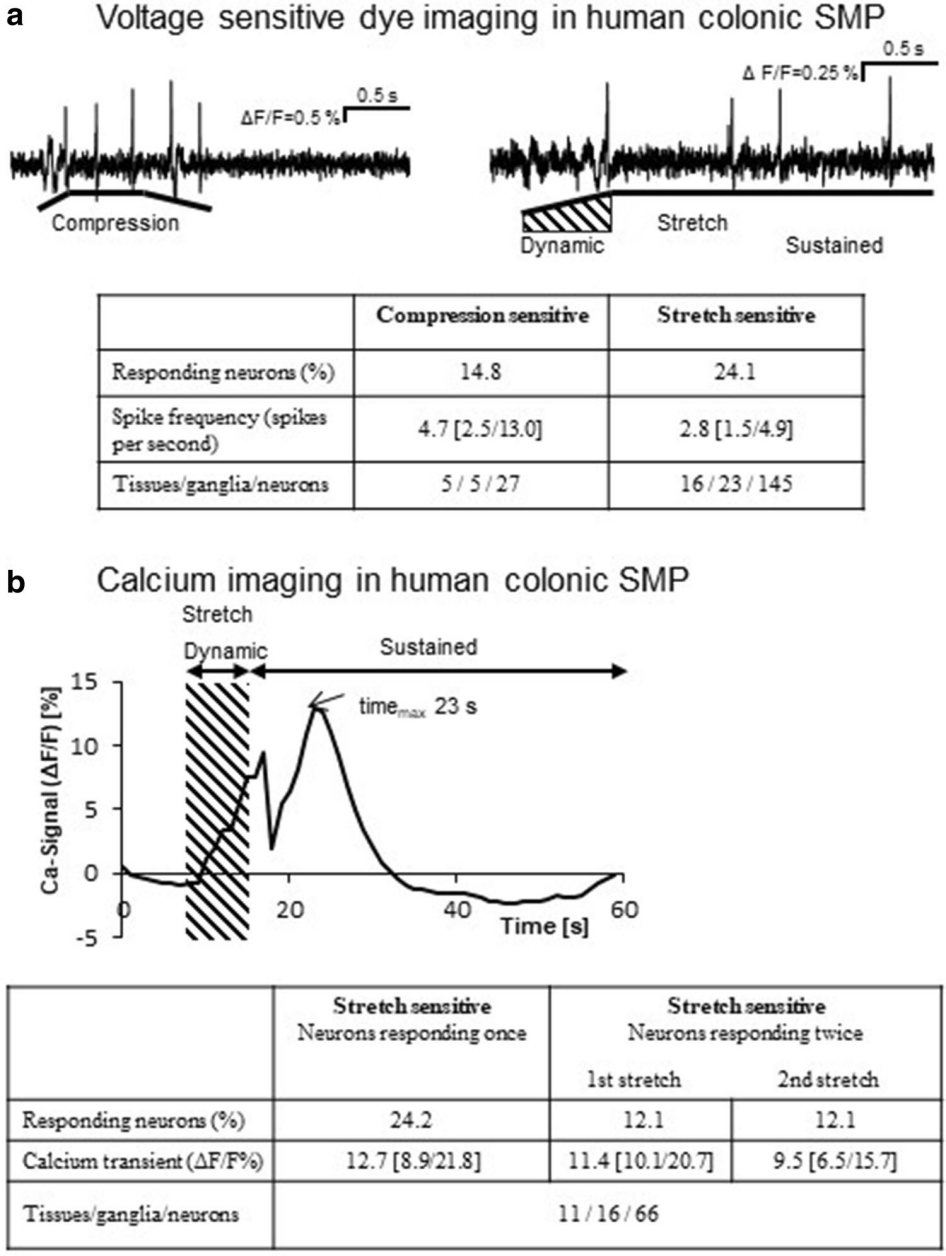
Neurons of human colonic submucous plexus are compression and stretch sensitive. (**a**) The trace on the left panel shows an example of a neuron that fired action potentials during the compression stimulus (intraganglionic volume injection). The graphic below the trace indicates the course of the stimulus: the injection (600 ms) causes a compression of the ganglion which is released slowly when the injected volume diffuses out of the ganglion. The trace on the right shows a different neuron responding to a stretch stimulus. Firing occurs during the dynamic phase (hatched area) and throughout the sustained phase. (**b**) The trace in the upper panel illustrates an example of a [Ca^2+^]_i_ signal in response to tensile stretch during dynamic (hatched area) and sustained stretch periods. Stretch evoked [Ca^2+^]_i_ peaks occurred in 24.2% of all neurons, half of them responded a second time after a 20 min resting period. Peak values of the [Ca^2+^]_i_ signals did not differ significantly between the first and second stretch stimulus. *P* = 0.461, Wilcoxon Signed Rank test.

Due to the smaller size of ganglia it was more challenging to apply mechanical stimuli in preparations of the human ISMP. The number and size of ganglia and interganglionic fiber tracts is more limited than in porcine preparations. Additionally, we observed that small shifts between ganglion and pipette during the application caused changes in injection efficiency between consecutive applications. These limitations precluded repeatability studies although we were able to see neuronal responses to repeated applications. Nevertheless, we were able to record responses to compression by intraganglionic volume injection in a subset of neurons (Fig. 7a). It was also not possible to reliably apply stretch in human ganglia with the same technique as in porcine preparations. Often, the orthodontic wires would lose contact to the connective tissue during the stimulus application. Therefore, we used blunt tips of glass pipettes that were placed on connective tissue strands next to the ganglion. This technique allowed us to apply multiple stretch stimuli to individual ganglia. Because the contact between glass tips and tissue varied over time, the stretch stimuli were not reproducible. With this technique, we applied stretch at rates of 20–30 µm/s for 1,000 ms which led to a median length change index of 7.8 [5.3/1.0]% (n = 19 stretch in 4 ganglia).This induced action potential discharge in a subset of neurons (Fig. 7a) and we could see that neurons responded to consecutive stretch stimuli. Bursts of action potentials lasted on average 1.9 ± 0.9 s (n = 63 recordings). Because of the variability in the immediate spike discharge recorded with voltage sensitive dye imaging we wondered whether the response is more robust when recording rather slowly developing Ca^2+^ transients. In human ISMP colonic neurons EFS evoked [Ca^2+^]_i_ signals instantaneously upon the onset of the stimulus with a maximum of 76.8 [51.0/135.8]% ΔF/F 2 [2/13]s after the onset of the stimulus (11/16/66). In total 16 of these neurons (24.2%) responded to a stretch stimulus (stretch rate 10–17 µm/s; duration of the dynamic period was 4 ± 2 s, the period of sustained stretch lasted until the end of the recording period of 60 s; Fig. 7b). The tensile stretch stimulus led to a median increase in ganglionic area of 4.6 [− 0.7/8.3]% (n = 8 tissues/10 ganglia). The [Ca^2+^]_i_ peak of 12.8 [8.9/21.8] % ΔF/F was reached 19.0 [13.0/27.8] s after stimulus onset. Almost half of those neurons (12.1%) showed similar [Ca^2+^]_i_ transients after a second tensile stretch stimulus 20 min apart. The amplitude of the [Ca^2+^]_i_ transients (Fig. 7b) as well as the time to reach [Ca^2+^]_i_ peak (first 14.0 [10.0/27.8] s vs. second stretch 12.0 [4.0/32.5] s; *P* = 0.547; data not shown) were similar.

## Discussion

With this study, we confirmed our hypothesis on the presence of MEN in the SMP of the porcine and human colon. So far, studies on SMP of the pig focused on the electrophysiology of neurons as revealed by intracellular recordings^37^ or the expression of transmitter and neuropeptides^8,38–42^. Although the pig is frequently advocated as a model for humans^31–36^, there are surprisingly few original studies which back up this notion. Our findings address one important aspect, which is a comparative study of sensory neurons in the ENS of the two species. We focused on the ISMP as a start as this may be relevant to better understand sensorimotor dysfunctions in diarrhoea or constipation.

Our data show that the ISMP of the porcine and human colon contains neurons sensitive to mechanical stimuli such as compression and stretch. Slight differences between stimulus parameters to distort ganglia were not due to species differences, but rather to specific tissue characteristics, such as higher proportion of fat, larger ganglia and stronger connective tissue in the pig compared to the much smaller ganglia and very loose connective tissue in human preparations (personal observation). In our study, we found 7.9% of the total neurons in the proximal and 10.4% in the distal colon of the pig being compression sensitive; the proportion in the human ISMP was with 14% comparable. It is striking that the proportion of myenteric compression sensitive MEN in guinea pig (12%) and mouse colon (14%) are very similar^14–16,43^.

In addition to compression, the sensitivity of myenteric neurons to tensile forces have been also shown^16,22,23^. Moreover, recordings of ion secretion in response to tissue distensions suggested the existence of stretch activated ion channels on enteric neurons other than capsaicin-sensitive neurons^27^. The stretching of porcine ISMP ganglia activated around 7.7% of the total neurons in the proximal and 6.3% in the distal colon. In human tissue between 12 and 24% were stretch sensitive. The stretch stimulus used in the current study led to changes in neuronal cell surface area, which was on average increased by 16.3% in proximal colon and 18.0% in distal colon of the porcine tissue samples. This is comparable to the results of stretch experiments in guinea pig distal colon with a change of neuronal surface of 15% (data not shown). The degree of in vivo distortion of guinea pig submucosal neurons has been investigated in fully contracted, spontaneously contracted, moderately distended and fully, physiologically distended ileum, reflecting changes in neuronal cell shape and size occurring under physiological smooth muscle contraction and relaxation, calculating a neuronal area increase of 17.9%^18^. Taking this into account, the stretch applied in the current study led to comparable results and therefore can be termed physiological. However, it should be noted that the experimental setup used only allowed detection of distortion in vertical and horizontal direction (X- and Y-axis). An additional undetected change in neuronal size and shape in depth (Z-axis) needs to be considered. We detected a positive correlation between strength of the applied stretch stimulus and neuronal activity of porcine submucosal neurons in the proximal and distal colon. Importantly, we found neurons located in the same ganglia undergoing similar distortion and hence cell surface area changes, which did not respond to the stimulus. This led us to conclude that only a subpopulation of ISMP neurons are mechanosensitive. Furthermore, this finding emphasized that the response was specific. Similar positive relations between stimulus strength and neuronal activity have already been shown for compression stimuli (intraganglionic volume injection and von Frey hair probing) as well as for stretch stimuli^14,16,19^.

To what extent porcine MEN may show adaptive response behaviour during prolonged deformation needs to be investigated in future studies. Our human Ca^2+^ imaging data seem to indicate that stretch sensitive neurons exhibit slowly adapting behaviour.

Immunohistochemical staining of submucosal MEN of the porcine proximal and distal colon for ChAT and SP immunoreactivity showed no significant differences between the intestinal regions investigated regardless of whether tensile or compressive forces were applied. A study performed in the porcine colon revealed that the proportion of enteric ISMP neurons immunoreactive for ChAT was 43%, 66% of those also expressed SP, and no neurons solely expressed SP^8^. Our immunohistochemical staining of MEN mainly reflected these data, and this agrees with our previous results in the guinea pig ileum and stomach, where MEN also did not show a particular neurochemical code^14,16^. Due to the fact that the majority of ISMP neurons project into the mucosal epithelium^8,9^, we could speculate that those cholinergic MEN are most likely to be involved in the regulation of secretory functions.

With regards to the proportion of NOS immunoreactive neurons among all neurons, we detected 10.3% and 29.5% NOS immunoreactive neurons in the proximal and distal colon. As shown for cholinergic neurons, the proportion of nitrergic MEN reflects the overall proportion of NOS neurons. However, in the distal colon a significantly higher proportion of identified compression and stretch sensitive MEN showed NOS immunoreactivity compared to the MEN in the proximal colon. This finding may be related to the larger amounts of water absorption that occurs in the distal colon which results in increasing viscosity and harder stool consistency. It seems plausible that the nitrergic MEN in ISMP of the distal colon decrease secretion as NO has been shown to inhibit release of excitatory transmitters presynaptically^44^. This would also mean that the NOS-expressing MEN rather project within the ISMP and do not project to the mucosa as NO increases secretion by a direct activation of epithelial cells^45^. It is noteworthy, that stretch sensitive MEN in the guinea pig gastric myenteric plexus were also mostly nitrergic^16^.

While the majority of MEN were activated by one mechanical stimulus only, a small population of porcine submucosal MEN is able to respond to both, stretch and compression. 3 out of 34 porcine ISMP MEN responded to both mechanical stimuli. This agrees with the data from other species and gastrointestinal regions^16,43^. This finding reinforces the translational aspect of our MEN concept across regions and species.

Is the pig now a good model for humans when it comes to the properties of MEN? The short answer is yes, because the proportion of MEN as well as the response characteristics are very similar. In our hands, this rather reflects the conservation of crucial pathways in the ENS across species. Previously, we have already observed similar behaviour of MEN irrespective of whether we recorded from human, guinea pig or mouse myenteric neurons^14,16,23^. In this respect, the pig is just another model and does not seem superior to the guinea pig, in particular since there are large differences between the anatomy of porcine and human colon. In addition, ganglia in the ISMP have a vastly different morphology and size; while ganglia in the human ISMP contain on average 8.4 ± 6.1 neurons per ganglion^46^, the corresponding number in the pig was 76.8 ± 64.7 and 76.1 ± 57.6 neurons per ganglion in the proximal and distal colon, respectively. In conclusion, depending on the scientific question(s) to be answered the pig can serve as a good model for the human gastrointestinal tract but critical aspects regarding differences also mentioned in this study need to be kept in mind.

## Material and methods

### Human tissue samples

Studies were performed using macroscopically normal surgical intestinal specimens of human colon obtained from 27 patients [11 female, 16 male, mean age 66 years (range 35–84 years)] undergoing abdominal surgery at the Medical Clinics in Freising and Erding as well as the Medical Clinic of the Technical University of Munich. Diagnoses that led to the surgery were as follows: colon carcinoma/adenoma (13/4), diverticular disease (5), and 1 each for Crohn’s Disease, colitis, stoma reversal, anorectal melanoma, intestinal polyps. Samples were taken from macroscopically normal, unaffected areas as determined by visual inspection by the pathologists. After removal, the surgical specimens were placed in cold aerated sterile HEPES-Krebs solution containing in mM: 135 NaCl, 5.4 KCl; 1.0 Mg Cl_2_, 1.2 NaH_2_PO_4_, 1.25 CaCl_2_, 12.2 Glucose, and 3 HEPES (all from Sigma-Aldrich Chemie GmbH, Darmstadt, Germany) and 10 mL/L antibiotic–antimycotic mix (mg/L: 25 amphotericin B, 107 U/L penicillin G, 10,000 streptomycin in physiological saline; CCPro, Oberdorla, Germany). They were immediately transported to the laboratory for experiments. Under continuous superfusion with ice-cold carbogen-aerated Krebs solution (pH 7.4), the surgical specimens were microscopically dissected by removing the mucosa and the muscular layers to obtain a whole mount preparation of the ISMP. The final preparations (10 × 20 mm) were pinned on silicone rings^47^. The use of nifedipine or nicardipine to minimize muscle movement during our recordings was not necessary, because the preparation used was mainly the ISMP without muscle layers.

### Porcine tissue samples

Intestinal tissues were obtained from hybrid pigs (female line German Large White × German Landrace; male line Pietrain) of both sexes, either with an age of 24 weeks (age group 1) or from pigs aged between 7 and 12 weeks (age group 2). All animals were either electrically or percussively stunned and exsanguinated by cutting their throat. The abdomen was opened and the GIT was quickly removed. Proximal and distal colon were separated: tissue samples of the proximal colon were gained 10 cm distal to the caecocolic junction, whereas tissue samples of the distal colon were taken 30 cm proximal to the anorectum. Samples were further dissected in carbogen aerated (95% CO_2_, 5% O_2_; pH 7.40) Krebs solution containing (in mM) 117 NaCl, 4.7 KCl, 1.2 MgCl_2_, 1.2 NaH_2_PO_4_, 25 NaHCO_3_, 2.5 CaCl_2_ and 11 glucose. The mucosa was gently removed to avoid damage to the ISMP lying beneath. Subsequently, all intestinal layers except from the ISMP were carefully removed and the prepared ISMP was pinned onto the rectangular opening (2 × 1 cm) of a silicone ring, which was placed in a recording chamber, continuously perfused with 37 °C carbogen aerated Krebs solution containing (in mM) 98 NaCl, 1.2 MgCl_2_, 1.2 NaH_2_PO_4_, 20 NaHCO_3_, 2.5 CaCl_2_ and 11 glucose with a perfusion rate of 10 mL/min.

### Ultrafast neuroimaging technique

An ultrafast neuroimaging technique was combined with a voltage sensitive dye to detect action potential discharge from enteric neurons as described previously^43,48^. Initially, the recording chamber containing the preparation was placed on top of an inverted microscope (Olympus IX71; Olympus Corporation, Hamburg, Germany). Individual ganglia of the ISMP were stained by intraganglionic injection of 20 µM Di-8-ANEPPS (1-(3-sulfanatopropyl)-4-[beta[2-(di-*n*-octylamino)-6-naphthyl]vinyl] pyridinium betaine; Thermo Fisher Scientific) dissolved in Krebs solution containing DMSO and Pluronic F-127 with a microinjection glass pipette (Science products, Hofheim, Germany). Staining did not affect the electrophysiological properties of enteric neurons^12^. Preparations were illuminated using a green high-power LED (LE T A2A true green (521 nm) 700 mA; OSRAM GmbH, Munich, Germany) combined with a filter-set containing a 525/15 nm bandpass excitation filter (AHF Analysentechnik, Tübingen, Germany), a dichroic mirror with a separation wavelength of 565 nm and a bandpass filter with a spectrum of 560/15 nm (AHF Analysentechnik). Due to high light intensity needed for appropriate signal to noise ratio a 40 × oil immersion objective lens (UApo 40 × OI3/340 Oil NA 1.35-0.5; Olympus Corporation) was used. For experiments with porcine tissue, changes in fluorescence intensity were recorded by an ultra-fast complementary metal oxide semiconductor (CMOS) camera system (256 × 256 pixels DaVinci-1K; RedShirt Imaging LLC) with a framerate of 1.25 kHz. The combination of CMOS camera and 40 × oil immersion objective lens resulted in a spatial resolution of 2.2 µm^2^ per pixel. Fluorescent signals were recorded and further analysed by using the Turbo SM 64 software (RedShirt Imaging LLC; https://www.redshirtimaging.com). Neuronal viability was verified by applying 100 µM nicotinergic acetylcholine receptor agonist nicotine directly onto single ganglia by local pressure application (PDES-2lL; npi electronic GmbH, Tamm, Germany). Voltage sensitive dye imaging of human tissue was done similarly with the exception that we used a CCD camera with a spatial resolution of 80 × 80 pixels (NeuroCCD SMQ, RedShirt Imaging LLC) at a framerate of 1 kHz. In combination with an × 100 objective we achieved a spatial resolution of 4.8 µm^2^ per pixel.

### Ca^2+^ sensitive dye imaging

We used Ca^2+^ imaging technique to assess long-lasting changes in intracellular Ca^2+^ levels ([Ca^2+^]_i_). The methods and techniques have been previously described in enteric neurons^48,49^. Briefly, the human SMP preparations were incubated for 45 min at room temperature in the dark with the fluorescent calcium indicator Fluo 4-acetoxymethyl (AM) (Invitrogen, Karlsruhe, Germany) at a final concentration of 10 µM diluted in Krebs buffer containing 500 µM probenecid (Sigma-Aldrich) to prevent dye leakage. The staining solution was washed out for 10 min and the preparation was mounted in self-made recording chamber and continuously perfused with 37 °C Krebs buffer.

The recording chambers were mounted on an inverted epifluorescence microscope (Zeiss Axio Observer A1, Carl Zeiss, Jena, Germany) equipped with a high-speed monochrome camera (Zeiss AxioCam HSm) and software (Zeiss Axio Vision 4.8; https://www.micro-shop.zeiss.com/de/de/system/software+axiovision-axiovision+basissoftware-axiovision+software/) for acquisition and analysis. Fluo-4 AM was excited using a blue light emitting diode (LED) Luxeon III (3 W, 470 nm dominant wavelength, Philips Lumiled, Phillips, Hamburg, Germany) and the signals were detected with a filter cube F26-514 Bright Line FITC BP (excitation: HC475/35, dichroic: 499, emission: HC530/43, AHF Analysentechnik, Tübingen, Germany) using 20 × objective (A-Plan, NA = 0.25, Zeiss). The system measured relative changes in fluorescence (ΔF/F) of Fluo-4 AM monitoring changes in [Ca^2+^]_i_.

Depending on the stimulus, [Ca^2+^]_i_ transients were recorded for 30 s (transmural electrical field stimulation, EFS) or 60 s (mechanical stimulation) using a frame rate of 1 Hz. Under basal condition the variation in background fluorescent was ± 1% ΔF/F. A threefold standard deviation (3%) was defined as threshold for genuine cell activation^49^. Basal, non-stimulated [Ca^2+^]_i_ transients were recorded for 35 s and 0.5 Hz.

### Mechanical stimulation of ganglia

Enteric neurons were mechanically stimulated by either intraganglionic volume injection, simulating compression or bidirectional stretch of single ganglia by a self-manufactured, two-armed stretching tool, simulating stretch. Both stimuli were applied twice with 10 min in between to verify reproducibility of the responses. The compression stimulus was generated by injecting Krebs solution (the same that was in the perfusion system) for 500 ms into a ganglion with a micropipette connected to the pressure application system. The pressure of the system was set at 0.5 bar which does not correspond to the pressure reached at the ganglion level. Complete distortion of all neurons of the chosen ganglion was ensured by visual inspection. Each injection of experimental Krebs solution had a volume of 14.9 ± 6.5 nL. This technique has already been applied successfully in submucosal and myenteric neurons of the guinea pig stomach, ileum and distal colon as well as in the mouse small and large intestine^14–16^. The self-designed stretching tool, mainly consisted of two orthodontic wires (TRU-CHROME #E00011; Rocky Mountain Orthodontics) rounded at their tips and inserted into two glass capillaries. Movement of these wires with a fixed speed of 15 µm/s was generated and controlled by two motorized (Minimotor SA, 15/2-900:1; Dr. Fritz Faulhaber GmbH, Schönaich, Germany) micromanipulators (M-3333; Narishige International Limited) (see supporting figures 1 and 2). Starting from their initial positions on opposite sides of a ganglion, both wires moved equally apart perpendicular generating a stretch of this ganglion. This is referred to as bidirectional stretch.

Stretching was achieved in human ISMP in a comparable way with the exception that the small size of the ganglia required glass pipettes with finer-tapered tips.

### Immunohistochemistry

Immunohistochemistry was used to identify chemical code of porcine MEN. This set of experiments could not be performed in human tissue due to technical reasons.

For immunohistochemical characterization of MEN, porcine tissue preparations were fixed immediately after the neuroimaging experiments for 12 h at 4 °C in a solution containing 4% paraformaldehyde and 0.002% picric acid (Sigma-Aldrich). Afterwards, tissues were washed (3 × 10 min) in phosphate-buffer saline (PBS) and pre-incubated for 1 h in PBS containing 4% horse serum (Sigma-Aldrich) and 0.5% Triton X-100 (Sigma-Aldrich). Tissues were incubated for 16 h at room temperature in a solution containing the primary antibodies (rabbit anti ChAT, 1:1,000, Prof. Dr. Schemann; rat anti SP, 1:1,000, Fitzgerald Industries International, Acton, MA, USA; mouse anti NOS, 1:1,000, Santa Cruz Biotechnology, Inc., Dallas, TX, USA) and then washed three times in PBS and incubated for 2 h in a solution containing the secondary antibodies (Cy 5-conjugated donkey anti-rabbit IgG, 1:500; Cy 2-conjugated donkey anti-rat IgG, 1:200; Cy 3-conjugated donkey anti-mouse IgG 1:500; all from Dianova, Hamburg, Germany). In a final step, tissues were washed three times in PBS, mounted on glass object slides and covered with a solution of PBS (pH 7.0) containing 0.1% NaN_3_ and 80% glycerol (Sigma-Aldrich). Preparations were examined using an epifluorescence microscope and appropriate filters. Images were taken and analysed with a monochrome camera (XM 10; Olympus Corporation) combined with Olympus cellSens Standard Software (Olympus Corporation; https://www.olympus-lifescience.com/de/software/cellsens). To exclude nonspecific binding of the secondary antibody a control staining omitting the primary was performed. Specificity of the used primary antibodies was already tested and reported in former studies^50,51^.The specificity of the mouse anti nNOS antibody was determined by staining the tissues with four different anti nNOS antibodies (rabbit anti NOS from Alexis, mouse anti NOS from Becton Dickinson, sheep anti NOS from Chemicon and mouse anti NOS from Santa Cruz) revealing that all antibodies marked identical neurons with the antibody selected leading to the brightest staining.

### Data analysis and statistics

Raw data of neuroimaging experiments was analysed using the Turbo SM 64 2.1.0.0 or Neuroplex 10.1.2 software (RedShirt Imaging LLC; https://www.redshirtimaging.com/). For the experiments in porcine tissue we calculated the proportion of MEN and their burst frequency, defined as number of action potentials divided by overall duration of spike discharge. Statistical evaluation and graphic display were performed using GraphPad Prism 8.0.1 (GraphPad Software Inc., La Jolla, CA, USA; https://www.graphpad.com) or SigmaPlot 12.5 (Systat, Erkrath, Germany; https://www.systat.de/) and Igor Pro 8 (WaveMetrics Inc, Lake Oswego, USA; https://www.wavemetrics.com/software/igor-pro-8). Unless indicated otherwise, data are presented as median and [Q_0.25_/Q_0.75_] due to a non-gaussian distribution of most data. Determination of applied distances by using the stretching tool, as well as calculation of neuronal size and area change was performed using ImageJ 1.8.0 (National Institutes of Health, Bethesda, MD, USA; https://imagej.nih.gov/). To calculate the effect of mechanical stretch, stretch index for each neuron was defined as the difference (%) between the original neuronal area (corresponding to 100%) and the area after bidirectional stretch. The lower resolution of the camera that was used for the experiments with human tissue did not allow precise measurement of the area of individual neurons. Therefore, we defined a "length-change index" as the sum of the absolute values of % change in the longest direction of the ganglion and a direction perpendicular to this.

For the Ca^2+^ imaging experiments the maximum intracellular [Ca^2+^]_i_ increase relative to resting light level (%ΔF/F) was determined as parameter for cell activation for each stimulus. Further, the time interval (s) from beginning of a stimulus to the peak [Ca^2+^]_i_ responses was analysed. The number of neurons responding to EFS was taken as 100% to quantify the percentage of neurons responding to a defined stimulus. In the [Ca^2+^]_i_ imaging experiments, the % change of human ganglionic area induced by tensile stretch stimulus was calculated using ImageJ 1.8.0.

The Wilcoxon Signed Rank Test was used to test for reproducibility of stretch responses.

*P* values < 0.05 were considered as statistically significant and are indicated by asterisks. Statistical tests were applied as indicated in the figure legends. Within the figure legends, n numbers are given as numbers of animals/ganglia/neurons. For image analysis of immunolabelled tissue preparations Olympus cellSens Standard Software (Olympus Corporation) was combined with ImageJ 1.8.0.

### Ethical approval

Procedures for the work with human samples were approved by the ethics committee of the Technical University of Munich (project approval 5242/11; 1746/07). Informed written consent was obtained from all subjects, and the studies conformed to the standards set by the Declaration of Helsinki. Intestinal tissues from animals assigned to age group one (see section below) were obtained from pigs killed for food production in a local slaughterhouse. In case of animals assigned to the second age group protocols for killing of the animals were approved by the State Office for Consumer Protection and Food Safety, Lower Saxony (permit number: 33.8-42502-04–16/2272 and 33.9-42502-04-18/2752) according to the German Animal Welfare Law and supervised by the Animal Welfare Commissioner of the University of Veterinary Medicine Hannover, Foundation.

## Supplementary information

Supplementary Information.

Supplementary Video.

## Data Availability

Data supporting the findings of this work are available within the paper and its Supplementary Information files.
