# Supplementary material for: Compression and stretch sensitive submucosal neurons of the porcine and human colon
Source: Sci Rep. 2020 Aug 14;10:13791. doi: 10.1038/s41598-020-70216-6 (PMC7428018; doi:10.1038/s41598-020-70216-6)
Supplement: Supplementary file 1 — Supplementary Information. [file 41598_2020_70216_MOESM1_ESM.docx]

**Compression and stretch sensitive submucosal neurons of the porcine and human colon**

Running title: mechanosensitivity of porcine submucosal enteric neurons

Anna Katharina Filzmayer^1^, Kristin Elfers^1^, Klaus Michel^2^, Sabine Buhner^2^, Florian Zeller^3^, Ihsan Ekin Demir^4^, Jörg Theisen^5^, Michael Schemann^2^, Gemma Mazzuoli-Weber^1^*

^1^Institute for Physiology and Cell Biology University of Veterinary Medicine, Foundation, Hannover

^2^Chair of Human Biology, Technical University of Munich, Freising, Germany

^3^Academic Hospital Freising, Germany

^4^University Hospital Rechts der Isar, Technical University of Munich, Germany

^5^Visceral and Thoracic Surgery, Academic Hospital Erding, Germany

Anna Katharina Filzmayer and Kristin Elfers share first authorship

Michael Schemann and Gemma Mazzuoli-Weber are joint senior authors.

***Corresponding Author**

Gemma Mazzuoli-Weber

Institute for Physiology and Cell Biology

University of Veterinary Medicine, Foundation, Hannover

Phone: +49 511 856 7543

Email: gemma.mazzuoli-weber@tiho-hannover.de

**Supplementary information**

**
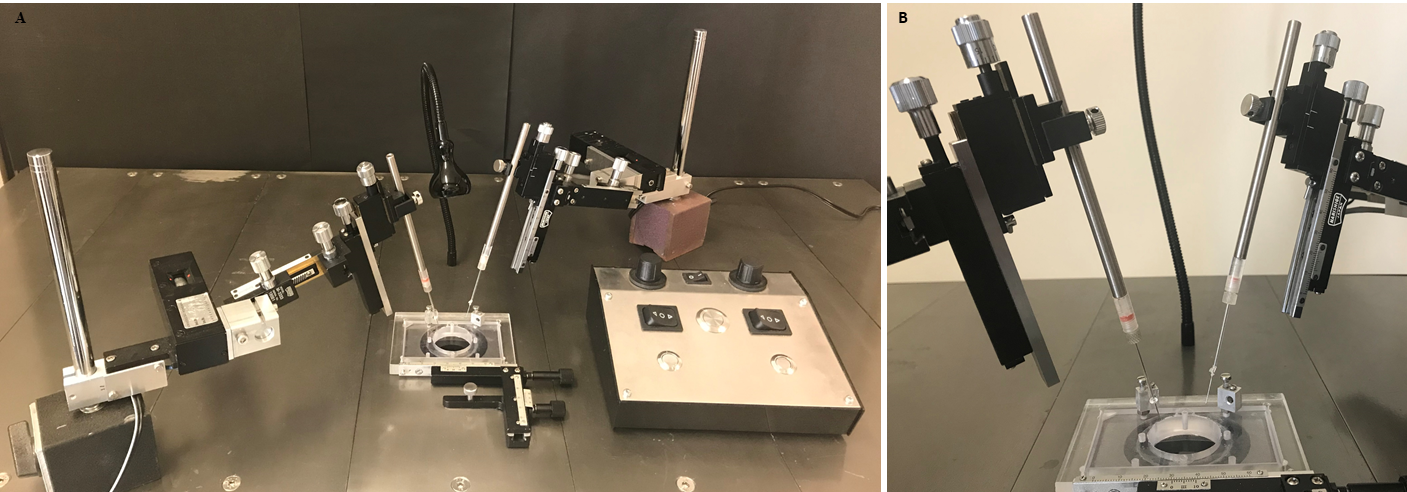
**

**
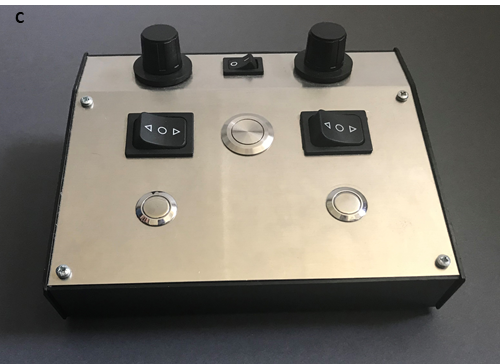
**

**Figure S1: Self-designed stretching tool allowing distention of single ganglia**. (A) shows the device including the controller and micromanipulators operating the two arms within the ultrafast neuroimaging setup. (B) shows how the two metal wires inserted in glass capillaries connected to the micromanipulators are positioned above the recording chamber in which the tissue is mounted during neuroimaging experiments. In (C) the controller is separately shown.


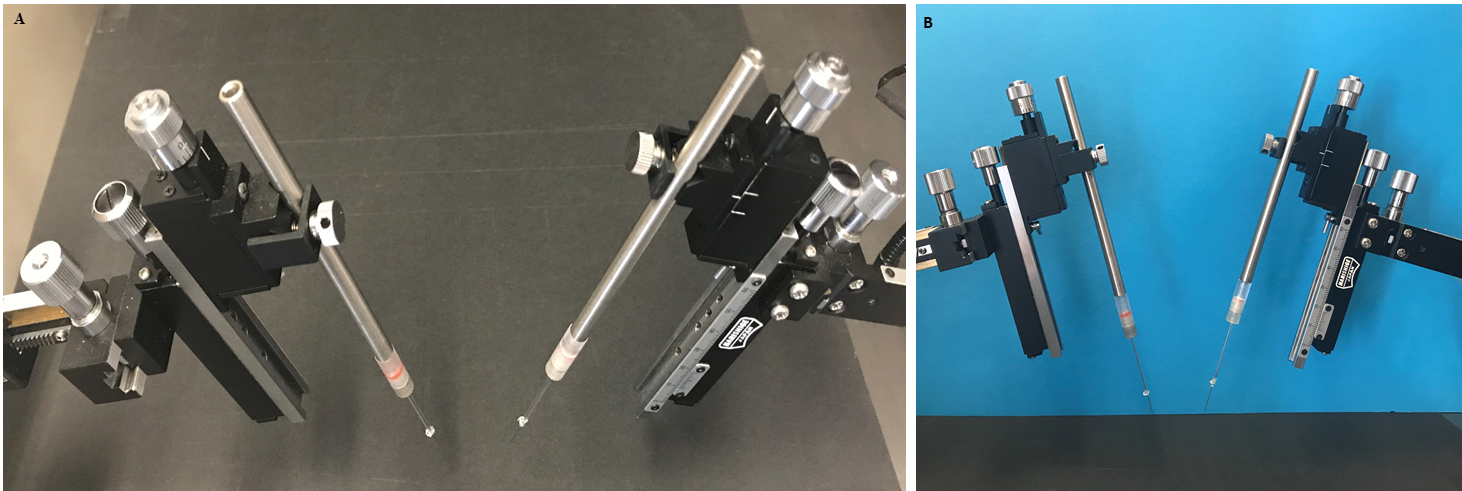


**Figure S2. Motorized arms of the stretching tool controlled by micromanipulators to which metal wires inserted in glass capillaries are connected.** (A) shows bird's-eye view of the device. In (B) the glass capillary holders with glued metal wires oppositely arranged are shown. The arms move simultaneously in opposite directions evoking an even bidirectional ganglionic distention.

**Supplementary Video 1.** This slow motion movie (original length is 1.6 sec) demonstrates deformation during compression by intraganglionic injection and spike discharge in one mechanosensitive enteric neuron. The outline of individual neurons can be recognize because Di-8-ANEPPS incorporates into the membrane. The responding neuron is a marked with white square and its corresponding trace is shown at the bottom. Once the movie starts a running red point indicates the elapsed time. The point is running from 0 to 1.6 s. At the beginning of the movie one can see the neuronal deformation due to the intraganglionic injection from the lower part of the ganglion. The overlay illustrates the color coded signals from the neuron; red color indicates discharge of action potentials. Shortly after the onset of the volume injection, the neuron fires a burst of action potentials.
